# Additive Manufacturing of Continuous Fiber-Reinforced Polymer Composites via Fused Deposition Modelling: A Comprehensive Review

**DOI:** 10.3390/polym16121622

**Published:** 2024-06-07

**Authors:** Muhammad Azfar Jamal, Owaisur Rahman Shah, Usman Ghafoor, Yumna Qureshi, M. Raheel Bhutta

**Affiliations:** 1 Department of Mechanical Engineering, Institute of Space Technology, Islamabad 44000, Pakistan; azfarjamal9@gmail.com (M.A.J.); owaisshah60@gmail.com (O.R.S.); yumna.qureshi@ensta-bretagne.org (Y.Q.); 2 Department of Logistics & Supply Chain Management, NUST Business School, National University of Science and Technology, Islamabad 44000, Pakistan; 3 Department of Electrical and Computer Engineering, University of Utah, Asia Campus, Incheon 21985, Republic of Korea

**Keywords:** additive manufacturing, 3D printing, fused deposition modeling, continuous fiber-reinforced polymer, fiber-reinforced polymer composites, FDM of CFRP

## Abstract

Additive manufacturing (AM) has arisen as a transformative technology for manufacturing complex geometries with enhanced mechanical properties, particularly in the realm of continuous fiber-reinforced polymer composites (CFRPCs). Among various AM techniques, fused deposition modeling (FDM) stands out as a promising method for the fabrication of CFRPCs due to its versatility, ease of use, flexibility, and cost-effectiveness. Several research papers on the AM of CFRPs via FDM were summarized and therefore this review paper provides a critical examination of the process-printing parameters influencing the AM process, with a focus on their impact on mechanical properties. This review covers details of factors such as fiber orientation, layer thickness, nozzle diameter, fiber volume fraction, printing temperature, and infill design, extracted from the existing literature. Through a visual representation of the process parameters (printing and material) and properties (mechanical, physical, and thermal), this paper aims to separate out the optimal processing parameters that have been inferred from various research studies. Furthermore, this analysis critically evaluates the current state-of-the-art research, highlighting advancements, applications, filament production methods, challenges, and opportunities for further development in this field. In comparison to short fibers, continuous fiber filaments can render better strength; however, delamination issues persist. Various parameters affect the printing process differently, resulting in several limitations that need to be addressed. Signifying the relationship between printing parameters and mechanical properties is vital for optimizing CFRPC fabrication via FDM, enabling the realization of lightweight, high-strength components for various industrial applications.

## 1. Introduction

As part of the fifth industrial revolution, AM is currently a hotly debated topic in scientific and industrial cultures, offering a fresh perspective on the uncharted contemporary world [1]. AM reduces design constraints, resulting in a shorter design and production cycle as well as a quicker fabrication procedure, which produces effective printed products, particularly for mass production and customized goods. It is a widely used processing technique, yet the strength of polymer-fiber composites produced by AM is far lower than that of conventional manufacturing methods such as vacuum-assisted resin transfer molding, injection molding, pre-preg layup, and vacuum bagging in an autoclave [2,3,4,5,6,7]. There are various techniques of executing AM; however, Stratasys introduced FDM for the first time, a layer-wise 3D printing technology to fabricate intricate geometrical objects. FDM is superior to other techniques due to its ease, flexibility, and cost-effectiveness [8]. Many studies have been conducted on polymer-based materials for 3D printing; however, printing structures with specific features, such as improved mechanical qualities and electrical conductivity, is not possible with neat polymers. Continuous fibers have a better load-bearing capacity than composites consisting of short fibers because they can transmit and hold loads within intact strands of fibers, which reduces the load delivered to and transferred by the polymer matrix. Merging reinforcement fibers with a polymer matrix reduces this limitation, resulting in a product with better structural strength that cannot be obtained from a single polymer material [9,10]. Using CFRPs as materials for 3D printing is a hot topic for researchers, as academic studies in the discipline of AM using CFRPs via FDM are still limited.

Parandoush et al. [11] presented a unique approach to laser-supported AM using a glass fiber–polypropylene matrix in combination with long fiber reinforcements. This technique employs laser-supported bonding and cutting to develop three-dimensional items. Bettini et al. [12] employed a unique method using FDM to produce continuous fiber-reinforced thermoplastics by making minor changes to a typical three-dimensional printer. Zhao et al. [13] and Ming et al. [14] suggested an FDM technique that uses ultraviolet light (UV) assistance and a dual-curing step to create long-fiber-reinforced composites. Ismail et al. [15] focused on the types of fibers and methods of reinforcement insertion employed for the AM of CFRP via FDM. A combination of FDM, void creation, and polymer sintering with respect to FDM 3D printing was analyzed. Heidari-Rarani et al. [16] created continuous fiber-reinforced thermoplastic (CFRT) composites using a novel extruder that was created and constructed for FDM 3D printers. Prusinowski and Kaczynski [17] assessed the tribological properties of fiber composites made by FDM using a novel head that fed the matrix material symmetrically. Aravind et al. [18] introduced a unique application of twisted carbon fiber filament and examined the impact on additively made materials using a single-nozzle configuration. Creating tension in the reinforcement, preparing the reinforcement surface, the feed rate, and the printing temperature are the main obstacles to creating a high-quality CFRP product. When using carbon fiber materials, load-oriented processes are especially important for curved, complex, and curvature-based geometries that require the creation of expensive molds. Kipping and Schüppstuhl [19] and Kipping et al. [20] underscored the significance of appropriate slicing and path-planning techniques. In their research papers, they presented a collection of methods designed to address issues such as highly curved and complicated geometries that are difficult to achieve with traditional techniques like tape laying and laminating, which also necessitate the expensive creation of molds. Zhang et al. [21] worked on the outcomes of using ultrasonic frequency on interfacial adhesion; additionally, they designed a novel FDM 3D printer with ultrasonic embedding. They found that interfacial adhesion and the bonding strength between CGF and PA6 were significantly improved.

Dickson et al. [22] evaluated the performance of long glass fiber, carbon, and Kevlar reinforcements in a combination matrix made with the additive manufacturing process of FDM. Markforged Mark One three-dimensional printing equipment was used to create these nylon composites. Akhoundi et al. [23] improved the mechanical characteristics of the parts made via an FDM extrusion-based 3D printer by maximizing the amount of continuous fiber yarn. In their study, Saeed et al. [24] assessed and compared the in-plane mechanical characteristics of long carbon fiber reinforcement with a polyamide matrix, produced by a Markforged Two three-dimensional printer, with expected values obtained by laminated-plate theory. Hedayati et al. [25] used polyglycolic acid suture yarn as the long reinforcement in combination with PCL scaffolds and printed the composite material using FDM. Kalova et al. [26] used the FDM technique to create a composite profile with long carbon reinforcements in combination with a matrix of onyx. In one of the research studies, Maqsood and Rimašauskas created porous and solid long carbon-reinforced composite models using 3D printing via FDM [27]. They also [28] used FDM 3D printing to produce a composite material containing long as well as short carbon fibers as reinforcements. The authors employed FDM to create four groups of specimens, as follows: PLA, PLA with short carbon fiber (PLA-SCF), PLA printed with continuous carbon fiber (PLA-CCF), and PLA-SCF printed with continuous carbon fiber (PLA-SCF-CCF) [27]. Liu, Xiong, and Zhou [29] presented the design idea for a CFRP that was manufactured by AM to enhance the performance of current products and promote innovations for upcoming needs. Billings et al. [30] used wood fibers, considered a flexible, renewable supply of cellulose, which were combined with bio-based PLA polymer to create sustainable, recyclable green composites that can be 3D-printed using FDM technology. To comprehend crucial material characteristics, the 3D-printed composites were thoroughly characterized. Uşun and Gümrük [31] examined the PLA thermoplastic-polymer-based AM of a CFRP with its excellent mechanical properties. Naik et al. [32] used an onyx matrix and continuous fiberglass reinforcement for creating the samples from the Markforged Mark Two composite three-dimensional printing system. Thermal gravimetric examination and an additional Fourier transform infrared spectroscopy test were undertaken. Table 1 exhibits various studies conducted on a wide combination of numerous fibers and polymers during the last 5 years (2019–2024). Caminero et al. [33] assessed how the build orientation, layer thickness, and fiber volume content affected the impact performance of FDM 3D-printed continuous carbon, glass, and Kevlar fiber-reinforced nylon composites. Chacón et al. [34] analyzed the impact of the build orientation, fiber volume content, and layer thickness on the mechanical performance of components made of continuous fiber-reinforced composites that were 3D-printed using a desktop 3D printer. Garcia et al. [35] compared the geometric features such as surface roughness, surface texture, flatness inaccuracy, and dimensional accuracy between a 3D-printed GNP-reinforced PLA composite and an upgraded PLA polymer (PLA-3D).

Hetrick et al. [53] investigated the effects of various fiber orientations (0, 90, 45), stacking patterns, and reinforcement patterns (unidirectional versus concentric) by 3D printing long Kevlar reinforcement in combination with an onyx matrix. The impact of each parameter was evaluated in relevance to the impact of energy absorption. Alarifi et al. [54] produced nylon composite material specimens at three different raster directions, i.e., 0, 90, and 45. The performance of the carbon and glass fiber reinforcements was evaluated using three-point flexural testing, with nylon serving as the matrix material. Wu et al. [55] in their research work used FDM to construct pure polyether sulfone (PES)- and continuous basalt fiber (BF)-reinforced composites. In-depth research was completed on the printing technique, temperature, abrasiveness, and interfacial bonding strength. In another study, the author of [56] employed the dissolving method utilizing the ASTM D 3171 standard approach which was used to estimate and determine the continuous carbon fiber (CCF) content of the composite. Maqsood and Rimašauskas [57] used FDM technology to create porous continuous carbon fiber-reinforced polymer structures. The porous structures were constructed with a single perimeter shell utilizing three distinct infill densities (20%, 40%, and 60%) and two distinct infill pattern types (triangular and grid). To enhance the mechanical qualities, Vatandaş et al. [38] thoroughly investigated several variables, such as the depth of each layer, printing temperature, speed, and fiber volume %. The study examined the performance of CFRTP composites from a different angle to the size of the reinforcing bundle. In their research, Caminero et al. [58] worked on the impact of layer thickness and fiber content upon the adhesion bonding. The bonding factor was evaluated by the FDM 3D-printing of long glass, and carbon, Kevlar reinforcements, and nylon matrix. The interlaminar shear behavior of FDM 3D-printed CFRP composites was studied by Yavas et al. [59] through a combination of computational and experimental investigation. The interlaminar shear strength (ILSS) of 3D-printed continuous and short CFRP composites was measured quantitatively using short beam shear (SBS) tests. In their research work, Touchard et al. [60] assessed the interlaminar and bond adhesion of the printed carbon fiber-PA6 matrix. Islam et al. [61] worked on continuous carbon-fiber-reinforced polymer composites using the FDM technique. However, voids developed between the layers as a result of the layer-by-layer deposition of materials, which reduced the interlaminar shear strength (ILSS) and other sections’ qualities. Mohammadizadeh and Fidan [62] presented a thorough investigation of the tensile characteristics of CFRPAM components. Antonios G. et al. [36] presented an experimental campaign that involved tensile testing on samples constructed of continuous carbon fiber and onyx. While continuous fiber samples with two, four, and six reinforcing layers out of a constant total layer number of sixteen were being investigated, the tidy onyx material was used as a reference. Representative samples were placed through micro-X-ray computed tomography measurements both prior to and during the test execution. When going from 0 to 4 continuous fiber layers, the tensile strength does not rise linearly, and the samples reinforced with 6 layers had a lower tensile strength than those with 4 layers. From the point of start until the point of final specimen rupture, the failure mode was also determined. This research may be viewed as a step closer to comprehending the real results of increasing the number of continuous fiber layers intended to strengthen the material, as well as the output of the FFF process when mixing short and long carbon/glass fibers.

**Figure 1:**
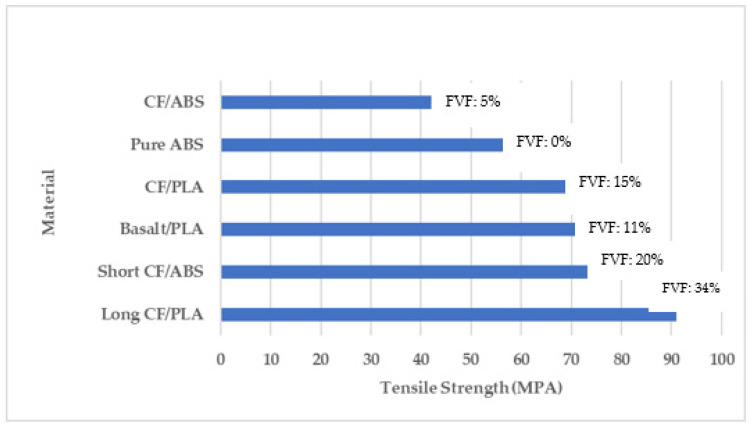
Tensile strength vs. material.

Figure 1 expresses the trends in tensile strengths against a specific strain rate for each type of material. The highest tensile strength (91 MPa) was achieved for long CF/PLA (34% fiber volume fraction (FVF)) at a strain rate (SR) of 2 mm/min, while the lowest tensile strength (42 MPa) observed was for CF/ABS (5% FVF) at an SR of 5 mm/min. This clearly demonstrates that increasing the strain rate results in the tensile strength of the material. On the other hand, the remaining four samples mentioned in Figure 1 illustrate closer values of tensile strength with short CF/ABS (20% FVF) having a tensile strength of 73.3%, basalt/PLA (11% FVF) having a tensile strength of 72%, CF/PLA (15% FVF) having a tensile strength of 69%, and pure ABS having a tensile strength of 56.5%, all at a strain rate of 1 mm/min. This depicts the concept that increasing the fiber volume fraction results in an increase in the tensile strength of the material as the fiber is the load-bearing phase of the composite material [63,64,65,66,67]. The composite samples were created using a dual nozzle 3D printer, and the final product’s tensile performance was assessed. Ding et al. [39] investigated the impact of mixed fibers on damage morphology and also the influence of the carbon/glass fiber layers on the strength and impact energy of the composite based on the fiber and matrix ratio. In their research, Saeed et al. [68] investigated the performance of long carbon reinforcement in combination with polyamide matrix composite specimens through tension and bending tests. Ibrahim et al. [69] investigated how the flexural characteristics of 3D-printed continuous wire polymer composites were affected by the wire treatment, wire volume percentage, and type of polymer matrix. Karimi et al. [70] primarily focused on continuous long fibers since continuous reinforcing fibers have a greater favorable influence on enhancing the strength of printed objects. The two primary fused filament fabrication mechanisms, the first one ex situ pre-preg and the second one in situ fusion, are thoroughly explained in this article. Additionally, the author provided pertinent illustrations of these systems with various reinforcing components. As per the author, all the mechanisms have pros and cons of their own, suggesting that further research and development are required to make 3D-printed FDM parts stronger than those made using conventional techniques. Another study discussed the effects of various continuous fiber and matrix polymer options on FRC performance. Along with reviewing the newest tools and techniques for creating FRCs, the author assessed the critical factors affecting FRC features. Additionally, the difficulties and flaws involved in 3D-printed fiber-reinforced composites were identified. Tuli et al. [71] and Hu et al. [37] covered the current possible technologies and their essential components based on the structure and substrate type of carbon fiber. The studies concentrated on the development of additively produced CFRCs using FDM and selective laser sintering (SLS). Additionally, the common uses and goals for CFRCs produced by additive manufacturing were explained in detail. A summary of the issues and obstacles that still remain was provided in the material, equipment, and software domains.

**Figure 2:**
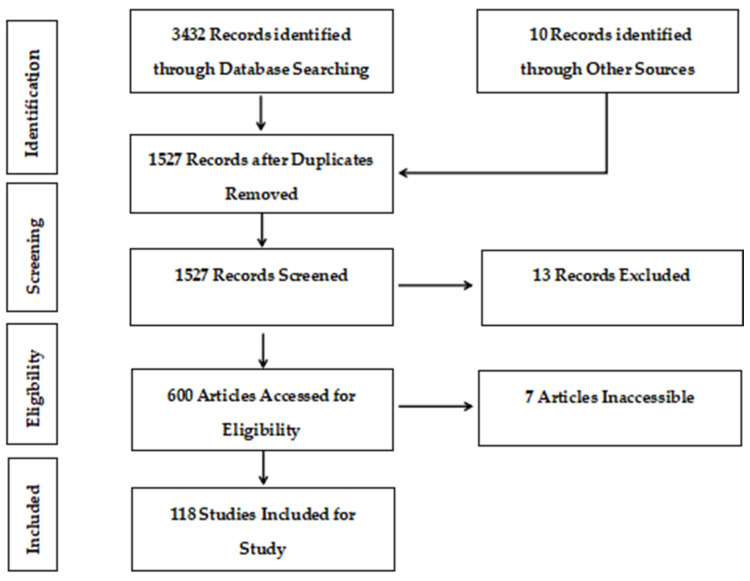
Prisma flow Diagram for Literature Review.

The database used for this study was Web of Knowledge out of which various papers relevant to the topic of the FDM of CFRP were extracted, reviewed, and summarized. The Prisma flow diagram expressed in Figure 2 displays the strategy utilized for the literature review. This review paper’s goal was to provide an insight into the AM of CFRP via FDM; moreover, it enlists the mechanical, physical, and chemical properties and printing material parameters that affect the fused deposition modeling process as well as the 3D-printed product. Various existing production methods of CFRP were discussed in detail. Also, the significance of the applications of 3D-printed CFRP in different industrial sectors was examined. Lastly, this study has also highlighted areas that need further research in AM of CFRP and discussed its future potential.

## 2. Fused Deposition Modelling of CFRP

**Figure 3:**
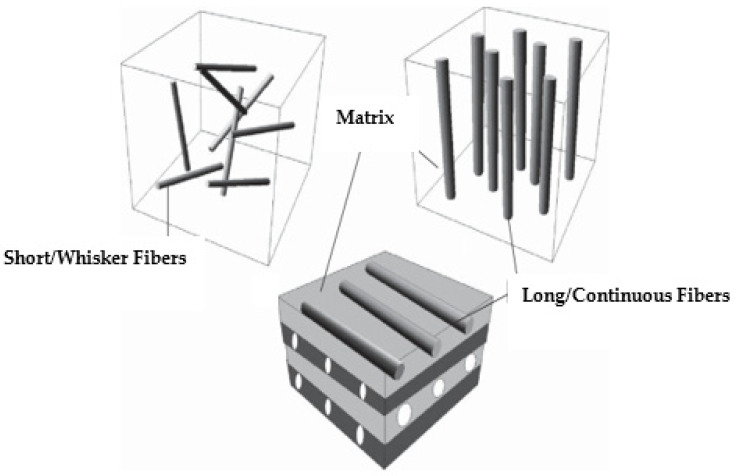
Illustration of SFRP vs. CFRP.

As part of the fifth industrial revolution, additive manufacturing is currently a hotly debated topic in scientific and industrial cultures, offering a fresh perspective on the modern world. There are few recent academic studies in the discipline of AM. AM processes are divided into several categories, such as electron beam processes, material adhesion, material jetting, laser melting, laser polymerization, and extrusion. The three primary materials used in 3D printers are powder, liquid, and solid. Each AM technique with respect to the material used is listed in Table 2. For the first time, Stratasys created fused deposition modeling (FDM) [72], a layer-wise 3D-printing technology that uses a computer numeric-controlled process in order to fabricate intricate three-dimensional objects by material deposition. The filament is deposited during the FDM 3D-printing process. A length of reel filament is deposited on the flat horizontal 2D plane, producing the first layer of the filament on the hotbed, after passing through a hot head that is heated at a temperature higher than the melting temperature of the material. Once the initial layer is printed, the next layer is assembled as the head advances along the Z-axis. FDM is the most important solids-based AM technology. Industries are finding it more and more attractive because of its ease of use, versatility, rapid prototyping, ease of changing materials, and better economics [23,73]. The most utilized polymer for FDM is thermoplastic because of its temperature characteristics. PLA, ABS, PP, PEEK, nylon, and polyamides are examples of thermoplastic filaments that can be used to make components for FDM [74]. Conversely, the significant properties of the reinforcing fibers include their corrosion resistance, high strength, and low weight. CFRP with enhanced characteristics is produced when these reinforcing fibers are effectively used in the standard 3D-printing process [75,76]. The most used reinforcing fibers are carbon fiber, glass fiber, Kevlar, onyx, basalt, etc. Fibers are basically of two types, i.e., short fibers and long/continuous fibers, also represented in Figure 3. 

### 2.1. Short Fiber-Reinforced Polymer Composites

The primary goal of accumulating short fibers to the polymer as reinforcement is to increase the printed part’s strength because printed components made entirely of polymers have poor strength, which limits their use in industrial applications. Short fibers are often mixed into a molten thermoplastic polymer to create the fiber-reinforced filaments utilized in the FDM method [77]. These tiny fibers are arranged randomly throughout the filament. Controlling the fiber orientation, the proportion of the mixture that is made of fiber, and the perfect fiber size are essential while making the fiber-accumulated filament to remove issues like the jamming of the extruder. These parameters have a major effect on the printed part’s qualities [78]. The stiffness, tensile strength, corrosive properties, fatigue strength, and damage tolerance of SFRCs are significantly better than unaltered polymers [79].

Setting up the most appropriate angle of raster, number of layers of reinforcement, temperature of extrusion, infill pattern, airgap, filament diameter, thickness of each layer, and layer deposition feed rate could all help to improve these mechanical properties when using SFRCs [80,81,82]. In one of the research studies, polypropylene thermoplastic was mixed with filaments containing different weight fractions of short carbon fibers using a Noztek filament maker. The parameters of the filament-making process were tuned based on the thermoplastic’s fiber composition.

The temperatures of the barrel and nozzle tip were adjusted according to the different weight percentages of carbon fiber. The optimum fiber-matrix adhesion, complete impregnation, and intended fiber distribution were guaranteed by a twofold extrusion procedure. After this process, the reinforced filaments’ short carbon fibers had an average length of 1.25 mm. The two-fold extrusion method and this parameter change were used to guarantee the homogeneous integration of carbon fibers into the filaments, enabling reliable 3D-printing outcomes [83]. Another researcher created novel reinforced 3D printing filaments, and the incorporation of recycled short carbon fibers into a polylactic acid matrix was evaluated. Filaments with a 5 and 10 weight percent recycled carbon fibers were generated. Additionally, filaments with the same concentration of washed virgin carbon fibers and pure PLA were constructed to examine the impact of the partially oxidized surface of recycled CFs on the adherence with the PLA matrix. The inclusion of CFs affects the mechanical and thermal characteristics of 3D-printed materials, as predicted [84]. Giani et al. [85] further described the utilization of recycled carbon fibers (rCF), which were produced by treating CFRP with pyro-gasification, to produce a thermoplastic-reinforced polymer for use in FDM. After optimizing the production process, filaments with rCF loadings of 10% and 5% of weight were created and examined. Sam-daliri et al. [86] described an optimized material extrusion 3D-printing process that used waste glass fiber-reinforced polypropylene (GFRPP) as feedstock. The impact of different glass fiber weight fractions (0%, 15%, 30%, and 40%) on the mechanical characteristics and printability of filaments and printed objects was also examined. By refining the parameters of the filament extrusion process, GFRPP feedstock filaments were produced. A filament with 40% fiber weight content achieved the maximum ultimate tensile strength (112 MPa). Furthermore, it has been demonstrated that filament quality may be improved, and the fiber aspect ratio can be decreased by continuous recycling.

Therefore, it is quite clear that short fiber reinforcements share advantages as well as a few disadvantages. The properties of the above-mentioned composite materials are clearly improved over pure polymer print, according to all the test findings; however, they still lag far below the strength characteristics of the composites that are traditionally made. The yield strength of the SFRP with the increase in fiber volume fraction is less than 500 MPa, whereas traditional composite production methods provide greater strength.

### 2.2. Continuous Fiber-Reinforced Polymer Composites

**Figure 4:**
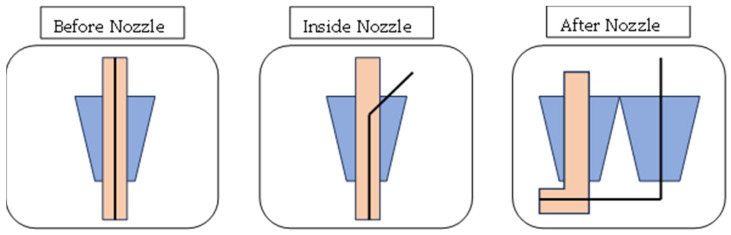
Manufacturing methods of CFRP with respect to position and time of fiber embedding.

The processes utilized to incorporate reinforcements into the thermoplastic polymer during the preparation of continuous fiber-reinforced polymer composites have an impact on the printed components’ mechanical characteristics. When considering the position and time of the fiber embedding, there are at least three distinct methods [87]. The three methods that researchers employ to embed fibers in the matrix and strengthen continuous fibers are shown in Figure 4, i.e., composite material formation prior, inside, and after the nozzle [15].

**Figure 5:**
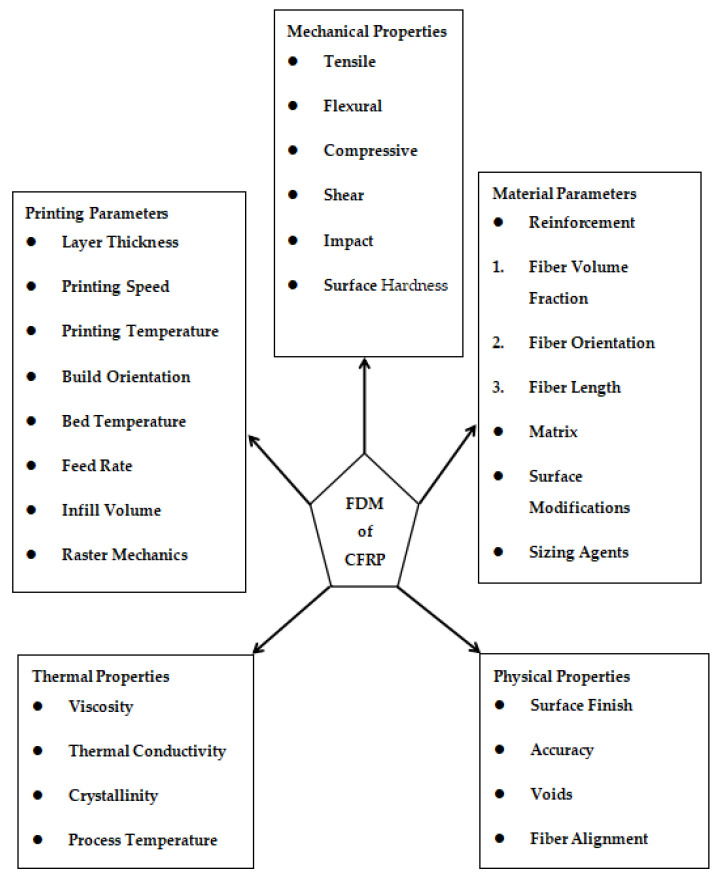
Parameters and properties of CFRP printing via FDM.

**Figure 6:**
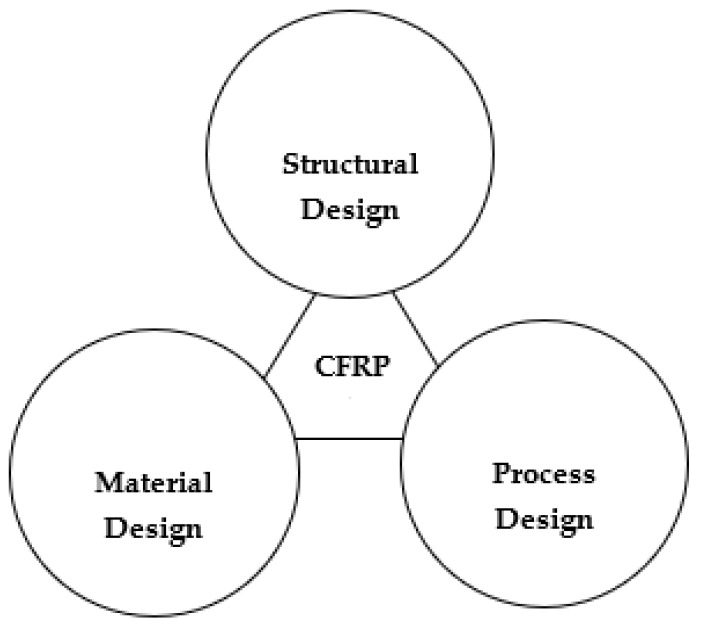
Design categories for FDM of CFRP.

CFRP could be 3D-printed using any of these three methods; the second method is also termed co-extrusion [88,89], and the third method is termed dual extrusion [22]. In the first method, the composite material is pre-fabricated before entering into the nozzle while the thermoplastic composite is brought to the FDM printer-head independently during the co-extrusion process. The thermoplastic filament melts in the heated nozzle, causing the resin to saturate the reinforcing fiber as it passes through. When the resin-coated fiber is sent through the nozzle and onto the printing platform, it adheres to the layer that came before it and solidifies [90,91]. In the case of dual extrusion, the thermoplastic resin and the reinforcements are extruded separately onto the printing plate using two nozzles in the dual extrusion process [92]. Since the fiber in CFRP is always oriented in the same direction as the printing, it is possible to modify its orientation. When printing, the stress that the reinforcing fiber exerts might assist in avoiding nozzle obstruction [7]. Carbon, glass, and Kevlar are the primary materials used in CFRP due to their better functions, which are beneficial in high-performance applications [12]. Before commercial printers that used the FDM technology to print continuous fiber-reinforced composites were introduced, researchers conducted a variety of studies to determine the best approach to insert continuous fibers into the thermoplastic matrix. Most investigations began with the creation of new extrusion heads and their integration with an existing FDM printer [63,93,94]. A commercial FDM 3D printer’s extrusion head was equipped with a preheating device designed by [89]. This method eliminated the need for an additional feeding mechanism by feeding the reinforcing fiber tow directly into the nozzle using a gearing system in conjunction with a stepper motor system to deliver the PLA filament. The reinforced filament deposits onto the platform layer-by-layer since the nozzle’s diameter is less than that of the thermoplastic filament. This occurred because the resin was forced into the nozzle by the solid filament as it melted into the resin. The tensile strengths of the continuous carbon fiber-reinforced thermoplastic (CFRTP) and the continuous jute fiber-reinforced thermoplastic composites, at 185.2 and 57.1 MPa, respectively, are higher than the PLA values. The use of these CFRTPs in engineering is becoming widespread. This is because of their unique qualities, which are hard to obtain in today’s engineering materials without sacrificing performance, and they exhibit qualities like low weight, extended life, high strength, and minimal maintenance. Many more studies have been conducted and currently work is being completed on the FDM of CFRP as there is significant capacity for research on this topic. Figure 5 expresses the parameters and properties relevant to the FDM of CFRP. Moreover, Figure 6 depicts the design domains linked to the FDM of CFRP.

### 2.3. Production Techniques of CFRP

Three commonly employed methods for the 3D printing of CFRP include compaction roller processes, co-extrusion, and dual extrusion [95,96].

The co-extrusion technique makes use of the polymer and the reinforcement, which are fed separately into the printing machine’s head. On the other hand, the dual extrusion technique employs two different nozzles to extrude the thermoplastic and fiber-reinforced filament independently [95].

**Figure 7:**
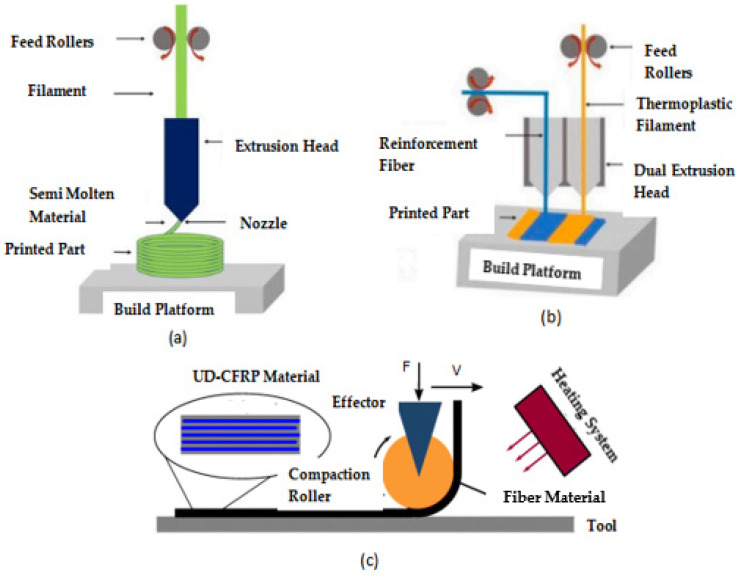
Production techniques of CFRP: (**a**) co-extrusion; (**b**) dual extrusion; (**c**) compaction.

In the compaction roller technique, a cartridge heater installed on the nozzle body provides the last support for the compaction roller. Internal bearings allow the compaction roller to freely move around the cartridge heater [96]. Figure 7 provides the visual representation of the above-mentioned three techniques.

### 2.4. Printing Parameters

Listed below are some of the most significant printing parameters along with their effect upon printed products and mechanical properties.

#### 2.4.1. Layer Thickness

The number of layers needed to print the object and, consequently, the printing time, are directly influenced by the layer thickness. Sample performance, interfacial bonding, mechanical qualities, and manufacturing precision are all impacted by layer thickness. A sample possessing superior mechanical qualities is one with a lower layer thickness; meanwhile, when the layer is thinner, printing takes longer. Consequently, it is necessary to choose the ideal thickness value that provides both respectable mechanical properties and a reasonable printing time. Optimal layer thickness is 0.05 to 0.4 mm; however, this varies depending upon the nozzle size [97].

#### 2.4.2. Printing Speed

At a slower printing speed, a stronger bond occurs between the filament and the continuous reinforcing fiber. The pace at which resin melts and the amount of time the filament stays in the extruder head may both be impacted by the printing speed.

As the filament spends less time in the nozzle at higher printing speeds, there is a decrease in pressure and impregnation time. While some studies have demonstrated that print speed had little effect on mechanical properties, most studies have shown that increasing print speed led to a loss of mechanical features [98].

#### 2.4.3. Printing Temperature

**Figure 8:**
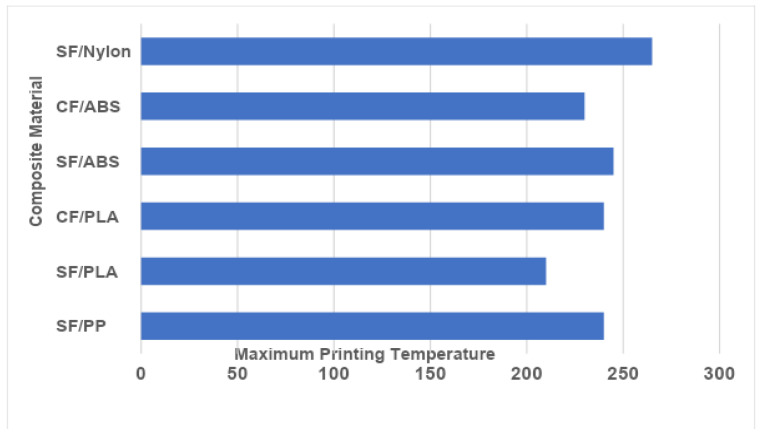
Maximum printing temperature vs. composite material.

As temperature affects the impregnation quality of reinforcing fibers, it is a critical element in CFRP 3D printing. The molten filament strengthens its bond with the composite as the printing temperature rises, enhancing its mechanical properties. At very high temperatures, however, printed composites lose their dimensional accuracy and aesthetic appeal. Therefore, it is important to select a temperature that will maintain the part’s mechanical properties and dimensional precision without sacrificing appearance. In certain cases, mechanical properties were unaffected by temperature [99]; this may be because of the limited temperature range for printing. Temperature-dependent increases in PLA/CF flexural strength and modulus were seen between 180 °C and 240 °C. On the other hand, the sample made at 240 °C lost its surface precision due to PLA melt overflow. Thus, 230 °C was the highest suggested printing temperature [100]. Figure 8 presents the maximum printing temperature for various CFRPs and SFRPs. For fiber-reinforced polymer composites, especially fused deposition modeling (FDM), selecting the ideal nozzle printing temperature is essential for obtaining the best possible material characteristics. A popular thermal analysis method for figuring out important thermal characteristics of polymers, such as their glass transition (Tg) and crystallization (Tc) temperatures, is differential scanning calorimetry (DSC). To guarantee the mechanical performance and thermal stability of the printed components, these data are crucial for setting the proper printing conditions. Paunonen et al. [101] employed a TA AQ20 differential scanning calorimeter (DSC) which was used to record the thermograms of unaged and completely aged composites in a nitrogen environment.

Using pliers, a thin test piece chip weighing between 3 and 5 mg of each material was cut, and it was then sealed within a typical aluminum pan with a cover. A consistent 10 °C/min heat–cool–heat temperature ramp was implemented from 0 to 200 °C. From the initial heating thermograms, cold crystallization, melting enthalpies, and temperatures were derived. Lee et al. [102] used a combination of experimental and computational methods to examine the impact of thermal convection within a commercial 3D printer on the thermal history and crystalline morphology of polyetheretherketone (PEEK). Polarized optical microscopy and DSC were used to examine the crystallinity of PEEK samples in relation to their thermal history. Additionally, the thermal history of the items during virtual 3D printing was assessed using finite element (FE) models of heat transport. It was discovered that PEEK’s high melting temperature causes quick melt cooling rates and brief annealing durations during printing, which results in a tiny crystalline morphology and a comparatively low degree of crystallinity. Rendas et al. [103] used various configurations and experiments were performed using DSC to evaluate the effects of thermal processing from various deposition sequences on the transition temperatures and crystallinity. To achieve this, samples were obtained half a radius away from the compression test specimens’ central layers. The DSC test was carried out at a maximum temperature of 400 °C for five minutes and heating/cooling rates of 10 °C/min. The heating curve provided the glass transition and melting temperatures, and the cooling curve provided the “hot” crystallization temperature.

#### 2.4.4. Build Orientation

**Figure 9:**
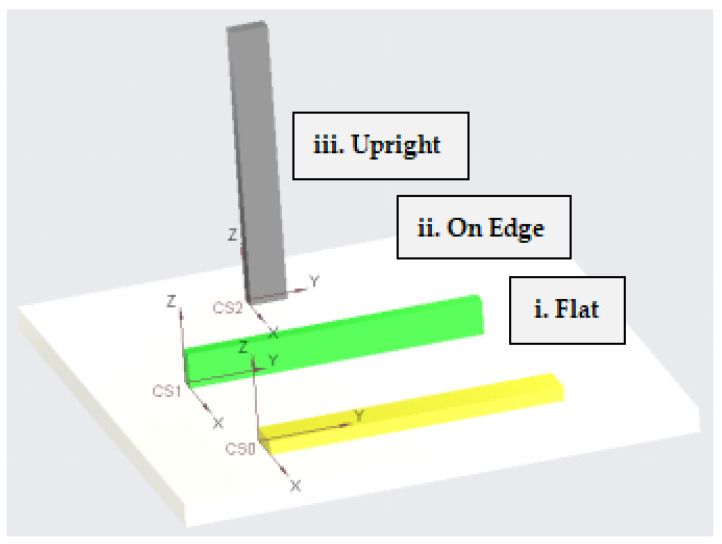
Build orientations: (**i**) flat; (**ii**) on-edge; (**iii**) upright.

For the 3D-printing of composites, three orientations are possible: upright, on-edge, and flat. Only a tiny number of scholars have examined this statistic, therefore more research is required. Using glass, carbon, and Kevlar fibers, Chacón et al. [34] printed nylon matrix composites that were reinforced on the edges and in a flat orientation. The results of the Charpy tests that were performed on the on-edge composites showed that they had a higher impact strength. The three different construction orientation types that were previously addressed are shown in Figure 9.

#### 2.4.5. Feed Rate

The filament feed rate determines how much material is put into the printing head in each length of time. It regulates the printing head’s internal pressure as well as the rate at which the melt material extrudes. PLA matrix-carbon reinforcement material was fed at different rates, varying between 60 to 160 mm/min. The parameters adjusted were as follows: 1.2 mm for hatch spacing, 0.5 mm for layer thickness, 100 mm/min for printing temperature, and 210 °C for printing speed. With increasing feed rates, the liquefier’s internal pressure as well as the contact pressure between the nozzle and the deposited layer rose, while the fiber content fell. The conflicting effects of these discoveries on the mechanical characteristics of the composite made the relationship between the filament feed rate and mechanical attributes unclear. To counteract these conflicting effects, a feed rate of 80–100 mm/min was advised [100].

#### 2.4.6. Infill Volume

The density and pattern of infill make up the infill volume. Kevlar, carbon, and glass-reinforced nylon matrix composites were printed by Mei et al. [104]. The composite’s infill patterns were triangular, hexagonal, and rectangular shapes. The number of fiber layers and concentric rings in each sample was the same. The number of concentric fiber rings and fiber layers was found to increase with the tensile strength and modulus. The paper claimed that a minor improvement in mechanical quality resulted from increasing the infill density. Moreover, the best mechanical properties were found in the hexagonal, rectangular, and triangle infill designs, in that exact order.

#### 2.4.7. Raster Mechanics

The deposited filaments, sometimes referred to as rasters, are stacked one on top of the other in the horizontal (XY) plane to form a layer. The material is deposited on top of the preceding raster in the through-thickness direction to form the first layer on the printed bed. The extrusion head and printing bed’s relative movements are controlled using computer numerical controls [105,106]. As they cool, the nearby deposited rasters combine to form a solid portion [107]. Although the material’s high temperature may aid with raster fusion, a prolonged exposure to high temperatures can cause the thermoplastic to sink because of gravity. The dimensional precision of a part is also influenced by the printing speed [108]. A slower printing speed allows for more ordered and accurate rasters since the nozzle moves more slowly, allowing the deposited rasters to be deposited layer after layer [109]. The pressure drop that controls the melt flow is influenced by speed, and therefore lowers the raster width. The ideal tensile strength angle in virgin polymers is 0 for a single raster angle, or in the direction of the tensile load. With the increasing raster angle, the mechanical performance decreases along with the printed structure’s load-bearing capability [109,110]. The tensile modulus does not change as the raster angle increases for angles greater than 45° [109]. This could be because, as opposed to material stiffness, the inter-raster welding stiffness determines the component stiffness.

### 2.5. Material Parameters

Mentioned below is a summary of some of the most crucial material parameters with respect to the FDM of CFRP.

#### 2.5.1. Reinforcements

Reinforcements are basically the fibers that are embedded into the matrix before printing. In the available literature, carbon, glass, Kevlar, and onyx are used as fibers. The important fiber characteristics listed below have an impact on the printed product’s characteristics:

##### Fiber Volume Fraction

Increased fiber volume fraction leads to better mechanical properties. FVF is an extremely potent factor that can boost strength in both tensile and flexural directions by up to 1000 MPa. Moreover, 25% FVF is the minimum required to reach a tensile strength higher than 600 MPa [111]. A continuous fiber composite 3D printer was also used to create curved fiber trajectories, which allowed for the creation of composites with different fiber volume percentages and stiffness. The stress field and the fiber trajectories were calculated and recalculated. By iterating through this process repeatedly until convergence was reached, optimization was accomplished. The optimized results yielded the strength and stiffness to be 1.6 and 9.4 times greater than the conventional ones [112]. It has been demonstrated that the impact strength rises with the number of fiber layers, raising the FVF [46].

##### Fiber Orientation

**Figure 10:**
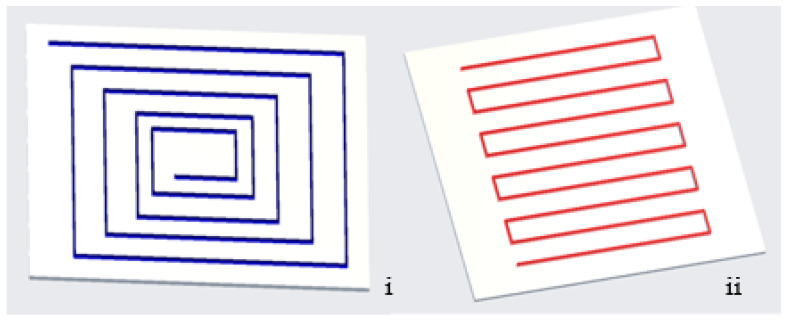
Fiber orientations: (**i**) concentric; (**ii**) isotropic.

There are two methods for printing the fibers, the isotropic and concentric. They can be printed at several angles (0, ±45, 90°) when in the isotropic form. Pyl et al. [113] altered the fiber orientations of the PA/CF composites to 0, 0/90, 0/90/±45, and ±45° to print the composites. The findings indicated that, in increasing order, composites having fiber orientations of 0, 0/90, 0/90/±45, and ±45° had the greatest tensile strength. The composites printed at the above-mentioned orientations all had almost the same strain to failure; however, the ±45° sample had a strain to failure that was roughly four times greater than the other samples. Araya-Calvo et al. [114] printed PA6/CF composites using isotropic and concentric infill patterns and obtained similar results. It was shown that concentric-patterned composites perform better after compressive and flexural testing. Dickson et al. [22] used reinforced glass, Kevlar, and carbon fiber with a nylon matrix. Comparing isotropic and concentric specimens, the composites exhibited a greater modulus and tensile and flexural strengths. Additionally, flexural tests showed that the concentric pattern composites bend more effectively than they tense. In several cases, an isotropic design produced better mechanical qualities; nevertheless, concentric patterns produced better mechanical characteristics in other investigations. Figure 10 shows both the concentric as well as isotropic fiber orientations.

##### Fiber Length

Zindani and Kumar [115] investigated how variations in carbon fiber length affected the flexural strength and fracture toughness. While 1 mm fibers had a higher flexural strength, 2 mm fibers were shown to have a better fracture toughness. It was stated that adding reinforcements would enhance the print quality [116]. FDM may be used in more applications by printing composites with ideal fiber lengths that have acceptable mechanical properties and are simple to manufacture. Researchers have examined how the length to weight ratio of the carbon fiber affects the mechanical characteristics of printed ABS resin components made with FDM technology [117]. Young’s modulus rose by just 5 weight percent, whereas tensile strength increased by 7.5 weight percent. Longer carbon fibers provide the most strength and stiffness.

#### 2.5.2. Matrix

A matrix in the form of a polymer or resin is used in combination with the reinforcement. Polymers such as thermoplastics are readily treated at temperatures below 300 °C and are used for 3D printing. When compared to metals or thermosetting polymers, the majority of thermoplastics used in FDM are low-quality varieties with low melting temperatures, a noticeable propensity to shrink during solidification, and poor mechanical performance. This often restricts the product’s use to prototypes only. Low-end FDM thermoplastics with low to intermediate mechanical and thermal characteristics are commonly used in typical applications. These include polypropylene (PP), polylactic acid (PLA), polycarbonate (PC), acrylonitrile-butadiene-styrene (ABS), and polyamide (PA, nylon) [118,119].

High-end thermoplastics that improve the mechanical performance can be utilized to create useful objects, such as polyether ether ketone or polyetherimide. However, a temperature of up to 350 °C is required for the correct processing of these thermoplastics, and this can only be achieved with specialized high-temperature equipment, high-performance heaters, and heat protection [67,120]. The composite notion, which includes strengthening the polymeric feedstock with a secondary phase, was enhanced using FDM to improve the substandard material qualities of ordinary thermoplastics [121,122]. Various fillers have been added to improve the mechanical and thermal characteristics of the polymeric matrix.

### 2.6. Mechanical Properties

The primary goal of FDM 3D printing using CFRP is to increase the strength and performance of the structures that are generated. Significant mechanical attributes include compressive strength, shear strength, impact strength, flexural strength, Young’s modulus, tensile strength, and many more. In Table 3, the record of the most prominent mechanical properties on which the researchers have worked for the last 6 years (2018–2024) is compiled. The record shows that tensile and flexural qualities have received the majority of research efforts; yet all of the previously listed mechanical properties still have potential for development. The data obtained indicate that the maximum tensile strength attained with unidirectional 3D-printed carbon fiber-reinforced nylon matrix specimens was 719 ± 46 MPa. The maximum elastic modulus achieved was 85 GPa using thermoplastic polyamide composite reinforced with carbon fiber. The highest flexural strength gained in a study was 310 MPa using continuous fiber-reinforced PLA filaments. The best flexural modulus achieved was 14.17 GPa using a concentric and equidistant reinforcement arrangement of carbon fiber-reinforced filament. Using unidirectional 3D-printed carbon fiber-reinforced nylon matrix specimens, the highest shear strength and shear modulus values were 48 MPa and 4 GPa, respectively. The best compressive strength acquired was 76.11 MPa using twisted carbon fiber filament. The highest compressive modulus observed was 2.102 GPa using a concentric and equidistant reinforcement arrangement of CFRP filament. The greatest impact energy achieved in terms of percentage was for Kevlar and glass fibers, when fully reinforced increased the impact strength for on-edge samples to 1233% for glass fibers and 777% for Kevlar.

### 2.7. Thermal and Physical Properties

When fiber reinforcement is added to a polymer, the volume of its crystalline structure rises. Fiber reinforcing, according to Sang et al. [64], decreased the crystalline temperature, which facilitated the formation of the amorphous phase to the crystalline structure. The carbon fiber in nylon worked as an effective nucleating agent for the crystallization process, according to Liao et al. [123], to lower the nucleation-free energy and promote the molecular chain’s organization into a crystalline phase. As a heat-absorbing thermal stabilizer, the fiber reinforcement raises the polymer’s breakdown temperature. On the other hand, this has a negligible impact on processing temperatures, namely the melting and glass transition temperatures. The capacity of the composite is increased by the addition of fibers in direct proportion to its fiber content and heat conductivity [124]. The enhanced heat conductivity, which promotes the transfer of residual heat from the earlier deposited raster to the more recent deposition, strengthens both the component and the inter-raster interaction. Due to fibers’ ability to improve heat transfer over neat thermoplastics, the fiber-reinforced filament eliminates weak areas by lowering localized high-temperature points and thermal residual stress [125]. Moreover, fibers improve dimension accuracy and avoid warping during production by reducing the coefficient of thermal expansion (CTE) [126]. The direction and degree of conductivity in anisotropic-reinforced fiber-reinforced thermoplastic is uncontrollable due to the random short fiber alignment. The fiber alignment technique was established to give the short fiber composite a controlled conducting direction [127]. The impact of various fiber designs on the viscosity of the polymeric matrix varies. According to Sang et al. [64], the viscosity of the molten matrix was proportionately increased by short fibers. Ivey et al. [118] concluded nozzle clogs may result from a high concentration of short fibers present in the matrix for some reasons, including fiber entanglement and insufficient back pressure to move the melted material through the nozzle. When it comes to short fiber reinforcing, PLA/short carbon fiber has a higher viscosity than short basalt fiber and is hence more printable than carbon fiber [64]. Furthermore, Zhang et al. [128] claimed that short carbon fiber created inter-raster gaps and decreased raster fusion by obstructing flow.

### 2.8. Defects

The possible drawbacks and shortcomings of 3D printing technology utilizing FDM are emphasized in this section. As anticipated, FDM/FFF offers several benefits in terms of material consumption, fabrication time, component complexity, and simplicity of use; they are covered in-depth in numerous papers. However, the quality and structural strength of the goods become a major worry in advancing the use of this technology from broad public usage and prototyping to large-scale production use, as well as to be assured of the integrity of the components even in a prototype [129]. This discussion offers debates and insights on the possible causes of 3D printer failures during part production and outlines the types and traits of probable faults that may occur in the parts that are generated. The destructive and non-destructive testing of AM components has shown mechanical defects such as lower resistance, anisotropy, and voids. The mechanical qualities of the constructed part, including voids with various characteristics, are influenced by the construction direction. Hernandez et al. [130] aimed to provide an expanded examination of the void form using FDM materials and X-ray computed tomography. Additionally, a relationship was established between the digital void measurements and the tensile mechanical characteristics. Well-established methods were used by Pace et al. [131] to identify the manufacturing flaws, and a thorough examination of the porosity distribution was provided for different onyx, onyx/carbon, and onyx/glass fiber-reinforced structure zones. In conclusion, total pore content exhibited a tendency to rise as the number of continuous fiber reinforcements increased. Also, differences in porosity along the printing direction were revealed. Mohseni et al. [132] investigated the effects of geometrical characteristics and printing settings on the void development of an automotive component. The development of non-invasive quality control for the imaging-based car-window holder will benefit from these results. The findings showed that the percentage of the voids reduced when printing speed and temperature were increased from 30 to 60 mm/min and 230 to 250 C, respectively. On the other hand, the vacancy fraction rose as the printing layer thickness grew (from 0.1 to 0.3 mm). It was also determined that in comparison to edges with an angle of 90 to 60, void generation is substantially higher in areas with curved surfaces and overhanging structures. To evaluate the effect of internal fractures on the mechanical performance of polylactic acid (PLA) samples, Mourad et al. [133] used FDM to 3D print the samples. Investigations were conducted into the shape, orientation, and placement of the defect throughout the sample gauge length, as well as the impact of process variables such as material color and infill %. Due to the porosity, the impact of the internal faults was more noticeable at a 100% infill rate as opposed to a 50% infill rate. Past performance is negatively impacted by voids because they impair the part’s dimensional, mechanical, and aesthetic qualities. Numerous factors, including the cross-sectional shape of the material tracks that have been deposited, the structure of the layer, and the layer-filling technique, affect the size and distribution of the voids. Sun et al. [134] examined a variety of void reduction techniques based on pre-deposition, in situ, or post-process methods. The key faults brought about by varying printing settings that might affect layer slicing and, in turn, affect the defect rate, are laid out in the suggested study by Ferretti et al. [135] The optimization method of choice was introduced together with proof of its applicability, indicating that a quality gain would result from it. The FDM process has advantages; however, it also has drawbacks, and some of these drawbacks include incomplete bottom layers, dangling strands, missing walls, pillowing, shifting layers, unfinished pieces, delamination of layers, burn marks, and uneven walls. The mentioned FDM manufacturing defects were examined and categorized by Gunaydin [136]. Additionally, methods for preventing certain manufacturing defects were also provided as solutions. Glinz et al. [137] examined the impact of various continuous fiber reinforcing materials and the quantity of continuous fiber-reinforced layers on AM build quality and tensile strength. Systematic print problems, such as asymmetric fiber positioning and inadequately pre-impregnated raw fiber material, were discovered by XCT examinations.

**Table 3:** Studies conducted on mechanical properties of CFRP via FDM (2018–2024).

Sr.#	Mechanical Properties of CFRP via FDM				
	Author	Year	CFRP Material	Mechanical Property Evaluated	Remarks
1	Saeed et al. [138]	2024	Fiber: Carbon Matrix: Nylon	Tensile Strength: 265 MPa Shear Strength: 56.47 MPa	The tensile strength of samples printed as a single piece in lap shear testing was found to be greater than that of samples with an adhesively bonded region of 12 mm and 25 mm.
2	Tuli et al. [71]		Fiber: Carbon, Glass, and Kevlar Matrix: Polyester, PLA, PP, Nylon, Epoxy, and LPDE	Max. Tensile Strength: 1600 MPa Max. Young’s Modulus: 92 MPa Max. Flexural Strength: 850 MPa Max. Flexural Modulus: 72 MPa	Highest tensile strength was found for Kevlar-LPDE composite. Highest flexural strength was found for carbon fiber-epoxy composite.
3	Vatandaş et al. [38]	2023	Fiber: Carbon Matrix: PLA	Tensile Strength: 1067.27 MPa for 3K CFRTP and 1090.22 MPa for 6K CFRTP Flexural Strength: 470.85 MPa for 3K CFRTP and 566.77 MPa for 6K CFRTP Flexural Modulus: 60.67 GPa for 3K CFRTP and 80.28 GPa for 6K CFRTP Shear Strength: 5.9 MPa for 3K CFRTP and 11.44 MPa for 6K CFRTP	The 6K CFRTP’s flexural strength rose by 20.37% when compared to the 3K CFRTP. The ILSS value increased significantly when 6K CFRTP samples were compared to 3K CFRTP samples.
4	Naik et al. [32]		Fiber: Fiber Glass Matrix: Onyx	Tensile Strength: 148.01 MPa	Triangular infill design with 0/90 fiber orientation absorbed max. impact energy, 14.90% and 8.98% higher than other patterns. For the same orientation, the maximum tensile strength was attained.
5	Islam et al. [61]		Fiber: Matrix:	Max. Shear Strength: 74.30 MPa	A novel robotic magnetic compaction force-assisted AM method was used for printing CFRP. The effects of z-threads and void-content control on the ILSS were investigated.
6	Zhang et al. [21]		Fiber: Glass Matrix: PA6	Tensile Strength: 521.5 MPa Flexural Strength: 397.1 MPa Flexural Modulus: 34.4 GPa Shear Strength: 62.3 MPa Impact Strength: 271.8 kJ/m^2^	Ultrasonography increased the interfacial adhesion between CGF and PA6, which strengthened the connection. The properties improve and the porosity of printed samples dramatically drops with an increase in frequency, from 4.52% to 1.33%.
7	Ding et al. [39]		Fiber: Carbon and Glass Matrix: Nylon	Tensile Strength: 585 MPa or carbon composite and 434 MPa for glass composite Impact Strength: 45 kJ/m^2^	The impact strength increased to 250%, but the tensile strength decreased to just 7%. With the same fiber content but different layouts, the tensile and impact strengths varied by 20% and 35%, respectively.
8	Alarifi [54]	2022	Fiber: Carbon and Glass Matrix: Nylon	Max. Young’s Modulus: 28.3 GPa Flexural Strength: 145.8 MPa	The composites exhibiting a 0-degree fiber raster orientation demonstrated the maximum flexural strength and modulus. The Dynamic mechanical analysis investigations revealed the nylon GF composite’s improved performance.
9	Ojha et al. [139]		Fiber: Kevlar Matrix: Onyx	Tensile Strength: 263 MPa Impact Strength: 90 kJ/m^2^	The impact strength of composite increased 3 times, whereas tensile strength enhanced 11 times. The experiment demonstrated that the mechanical characteristics of fibers are influenced by the loading direction.
10	Saeed et al. [24]		Fiber: Carbon Matrix: Polyamide	Tensile Strength: 524.66 MPa Young’s Modulus: 73.20 GPa	Due to a 35% increase in the fiber volume fraction from 29%, the hot-pressed samples showed an increase in tensile strength of 27% and an increase in elastic modulus of 11%.
11	Maqsood and Rimašauskas [57]		Fiber: Carbon Matrix: PLA	Tensile Strength: 162.9 MPa (grid infill), 152.62 MPa (triangular infill) Flexural Strength: 127.24 MPa (grid infill), 117.53 MPa (triangular infill)	The samples that were 3D-printed with a grid infill pattern and a 60% infill density showed the most endurance. In a triangular infill pattern with a 60% infill density, the greatest tensile and flexural strengths were determined.
12	Wu et al. [55]		Fiber: Basalt Matrix: Polyester	Tensile Strength: 54.72 MPa Flexural Strength: 83.34 MPa	The PES printed at 360 degrees Celsius has the highest tensile strength possible. Tensile and bending strengths of the PES/BF composite were found to be 217.06% and 87.96% greater, respectively.
13	Uşun and Gümrük [31]	2021	Fiber: Carbon Matrix: PLA	Tensile Strength: 544 MPa Flexural Strength: 310 MPa	Maximum tensile strength and maximum flexural strength were shown by CFRTP specimens with a 40% fiber content.
14	Hetrick et al. [53]		Fiber: Kevlar Matrix: Onyx	Impact Strength: 31 J	The lowest absorption of impact energy was observed for the specimens with fiber orientation at 90 degrees. The 45 angle-ply fiber orientation specimens had the maximum absorption of impact energy.
15	Maqsood and Rimašauskas [140]		Fiber: Carbon Matrix: PLA	Tensile Strength: 245.4 MPa Young’s Modulus: 27.93 MPa Flexural Strength: 168.88 MPa Flexural Modulus: 10.85 GPa	PLA-CCF composite had the greatest Young’s modulus and maximum tensile strength. The PLA-CCF specimen had a strength that was 7.84% more than the PLA-SCF-CCF specimen and 460% higher than the pure PLA and PLA-SCF specimens. The largest mean flexural stress value was found in PLA-CCF specimens. The PLA-SCF printed with CCF had the highest mean flexural modulus.
16	Maqsood and Rimašauskas [28]		Fiber: Carbon Matrix: PLA	Flexural Strength: 134.58 MPa Flexural Modulus: 12.26 GPa	The specimen’s micrographs after the flexural test showed that the cause for delamination was the composite’s poor and insufficient interfacial bonding, which led to gaps and separation between them.
17	Kalova et al. [26]		Fiber: Carbon Matrix: Onyx	Compressive Strength: 594 MPa	The experimentally determined critical force at composite profile collapse had a mean value of 3102 N, whereas the critical force, using FEM analysis, was 2879 N. There was only a 7% difference in critical force.
18	Mohammadizadeh and Fidan [62]		Fiber: Carbon, Glass, and Kevlar Matrix: Nylon	Max. Tensile Strength: 446.87 MPa	The tensile strength and elastic modulus of CFRAM components rose by up to 2231% and 17,206%, respectively. The printed component’s tensile strength was higher than that of aluminum 6061.
19	Touchard et al. [60]		Fiber: Carbon Matrix: PA6	Shear Strength: 399 MPa	The loading rate for double cantilever beam specimens was kept constant at 5 mm/min. The ultimate delamination length was around 85 mm, which predicted the ILSS.
20	Aravind et al. [18]	2020	Fiber: Carbon Matrix: PLA	Tensile Strength: 200.33 MPa Flexural Strength: 141 MPa Compressive Strength: 76.11 MPa	With a constant layer thickness of 1 mm and a 30.5% volume percent of carbon fiber, Tensile strength could reach 200.33 MPa, compression strength could reach 76.11 MPa, and flexural strength could reach 141 MPa.
21	Hedayati et al. [25]		Fiber: Polyglycolic acid Matrix: Poly (ε-caprolactone)	Tensile Strength: 79.7 MPa Tensile Modulus: 3.5 GPa	Young’s modulus improved to 775% and strength significantly increased to 374% with suture yarn having a content of 22 vol.%. Fiber degradation phenomena dominated the deterioration of the composite, which was 20 times greater.
22	Saeed et al. [24]		Fiber: Carbon Matrix: Polyamide	Tensile Strength: 603.43 MPa Tensile Modulus: 85 GPa,	Tensile strength and modulus were found to be much higher than those of unreinforced nylon specimens.
23	Yavas et al. [59]		Fiber: Carbon Matrix: Onyx	Shear Strength: 40.9 MPa (48 layers of CFRP) and 24.4 MPa (24 layers of CFRP and 24 layers of SFRP)	The ILSS was calculated using the first composite layup, (48 layers of CFRP) and the second composite layup, (24 layers of CFRP and 24 layers of SFRP). The first layup has better value for ILSS.
24	Ming et al. [14]		Fiber: Glass Matrix: Epoxy	Tensile Strength: 272.51 MPa Young’s Modulus: 8.01 GPa Flexural Strength: 299.36 MPa Flexural Modulus: 8.35 GPa Shear Strength: 34.06 MPa	Tensile strength and tensile modulus of 272.51 ± 5.12 MPa and 8.01 ± 0.45 GPa, respectively. Flexural strength and flexural modulus of 299.36 ± 6.16 MPa and 8.35 ± 0.18 GPa, respectively. Interlaminar shear strength of 34.06 ± 0.83 MPa was demonstrated.
25	Chacón et al. [34]	2019	Fiber: Carbon, Glass, and Kevlar Matrix: Nylon	Max. Tensile Strength: 436.7 MPa Max. Young’s Modulus: 51.7 GPa Max. Flexural Strength: 423.5 MPa Max. Flexural Modulus: 39.2 GPa	The findings demonstrate that flat samples outperform on-edge samples in terms of strength and stiffness Carbon composites had the greatest mechanical performance and maximum stiffness.
26	Heidari-Rarani et al. [16]		Fiber: Carbon Matrix: PLA	Tensile Strength: 61.4 MPa Young’s Modulus: 8.28 GPa Flexural strength: 152.1 MPa Flexural Modulus: 13.42 GPa	Composite samples were tested using quasi-static stress to assess the product’s quality. The testing findings showed a considerable improvement in PLA’s tensile and bending capabilities.
27	Ibrahim et al. [69]		Fiber: Carbon Matrix: PLA	Flexural Strength: 107 MPa Flexural Modulus: 6.2 MPa	The wire-reinforced carbon fiber matrix generated a maximum ultimate flexural strength with a 1.7% wire volume fraction. The flexural modulus of the reinforced samples was greater than the unreinforced samples.
28	Akhoundi et al. [23]		Fiber: Glass Fiber Matrix: PLA	Tensile Strength: 478 MPa Young’s Modulus: 29.4 GPa	With a configuration of several process printing parameters, a fiber-volume content of around 50% could be achieved. Suitable results for tensile strength and Young’s modulus were achieved
29	Mei et al. [104]		Fiber: Carbon Matrix: Nylon	Tensile Strength: 110 MPa Young’s Modulus: 3.491 GPA	The results demonstrated that the CF-printed composite had the greatest modulus and tensile strength. Concentric fiber rings and fiber layers are correlated with an increase in tensile strength and modulus.
30	Caminero et al. [33]	2018	Fiber: Carbon, Kevlar, and Glass Matrix: Nylon	Max. Impact Strength: 82.26 kJ/m^2^ (Carbon), 184.76 kJ/m^2^ (Kevlar) and 280.95 kJ/m^2^ (Glass)	Compared to unreinforced ones, fully reinforced (type C) tended to induce higher impact strength For Kevlar and glass, increasing the fiber %age from type A (partially reinforced) to type C tended to increase the impact strength. Type C on-edge samples outperformed flat samples in terms of impact efficiency.
31	Caminero et al. [58]		Fiber: Carbon, Kevlar, and Glass Matrix: Nylon	Max. Shear strength: 31.94 MPa (Carbon), 14.28 MPa (Kevlar) and 20.99 MPa (Glass)	The ILSS values decreased with layer thickness due to the increasing porosity. The ILSS values of continuous fiber-reinforced materials were higher than those of unreinforced samples. Carbon composites provided greater stiffness and the best interlaminar shear performance.
32	Pyl et al. [113]		Fiber: Carbon Matrix: Nylon	Tensile Strength: 719 MPa Young’s Modulus: 58.07 GPa Shear Strength: 48 MPa Shear Modulus: 4 GPa	The results showed that the elastic tensile strength of 719 ± 46 MPa, a strain to failure of 1.26 ± 0.09%, and an elastic modulus of 58.07 ± 1.86 GPa. A shear modulus of 4 GPa and a shear stress of 48 MPa were obtained.
33	Araya-Calvo et al. [114]		Fiber: Carbon Matrix: PA6	Flexural Strength: 231.1 MPa. Flexural Modulus: 14.17 GPa Compressive strength: 53.3 MPa Compressive Modulus: 2.102 GPa	For a volume ratio of 0.2444 carbon fiber, with a concentric and equidistant reinforcing arrangement: stress at a proportional limit of 53.3 MPa and a compressive modulus of 2.102 GPa were achieved. The flexural modulus was 14.17 GPa and the proportional limit was 231.1 MPa.

### 2.9. Failure Modes

Pollet et al. [141] evaluated how hygrothermal aging affects the Mode-I fracture toughness behavior of carbon/epoxy and glass/epoxy composites made via filament winding. Different winding angles (±0°, ±15°, ±30°, and ± 45°) were used to create cylinders (136 mm in diameter). While carbon/epoxy exhibits higher fracture toughness for the same winding angle and aging, glass/epoxy composites perform better in terms of peak load and strain energy release rate. The curvature and challenge of maintaining symmetry during the test results in more complicated fracture processes in the curved, double-cantilever beam samples than in the flat samples. Lambiase et al. [142] investigated how the deposition approach affects the inter-layer zone’s fracture toughness behavior in FDM 3D-printed components. To capture the fracture toughness behavior, double-cantilever beam specimens were created and evaluated, following accepted testing procedures. Linear layouts with alternative and monodirectional infill procedures were the tested conditions. Optical microscopy observations were used to cross-check the differences in the mechanical behavior of the samples. The findings showed that these components’ fracture toughness behavior was significantly impacted by the deposition pattern. For the 0° and 90° raster angles, monodirectional deposition techniques included a fracture toughness of 0.75 and 2.4 kJ/m^2^, respectively. Kizhakkinan [143] used an experimental design to explore the fracture toughness of PLA pieces manufactured using the FDM additive manufacturing technology. The filament orientations used for the compact tension (CT) specimens were 0°/90° and −45°/45°, and the printing rates varied from 20 mm/s to 60 mm/s. The linear elastic fracture mechanics method was utilized to determine the values of fracture toughness for every process parameter. The CT specimen that was produced at the fastest speed had the lowest fracture toughness rating, but it also had the maximum energy absorbed before failure. In comparison to the 0°/90° specimen, the −45°/45° CT specimen had a greater value of fracture toughness. To assess the mechanical behavior, tensile tests were also performed on part-level coupons and PLA filament. A brittle breakdown was seen in the FDM-printed tensile coupon. On the other hand, the PLA filament exhibited ductile behavior and a distinct plastic zone. In another study, Stamopoulos et al. [144] determined the inter-layer fracture toughness behavior of FDM components. To capture the fracture toughness behavior, double-cantilever beam specimens were used. To remove some of the restrictions on sample preparation—which is also impacted by human factors—a novel sample configuration was also suggested. To ascertain if the suggested approach was appropriate for addressing the crucial energy release rate in Mode-I crack opening, the two types of samples were compared. Optical and scanning electron microscopy ex situ measurements were used to examine the fracture zone properties.

Stamopoulos et al. [145] employed mechanical testing and numerical modeling which were used to assess the impact of porosity on the shear mechanical characteristics of unidirectional carbon fiber-reinforced plastic composites. Within this context, specimens of carbon fiber-reinforced plastic with four different porosity levels were subjected to the V-notched rail shear test technique. The progressive damage model and the virtual crack closing technique were the two finite element approaches used to simulate this specific mechanical test. The benefits and drawbacks of the two numerical techniques were evaluated, showing satisfactory agreement with the outcomes of the mechanical testing.

## 3. Applications

**Figure 11:**
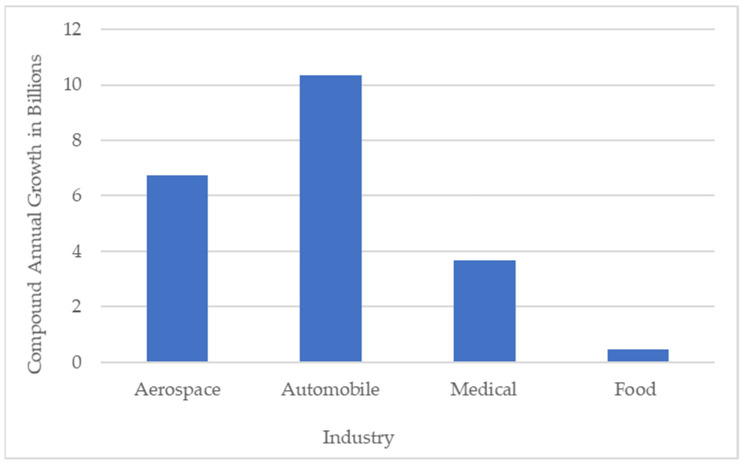
Industry vs. expected compound annual growth until 2026.

There are various applications of the 3D printing of CFRPs targeting various industrial sectors such as aerospace, medical, automotive, food, defense, construction, etc. Saeed et al. [134] assessed the fatigue life performance of polymer composites that were 3D-printed using the FDM process. The study provided an estimate of the component’s safe service-life during operation and fatigue life assessment is crucial for developing components for the aerospace, medical, and automotive sectors. This work attempted to close the knowledge gap on the fatigue behavior of 3D-printed polymer composites. The 3D-printed polymer composites underwent fatigue testing under various loading scenarios and static (tensile) testing to ascertain their ultimate tensile strength. The 3D-printed materials in this study were also subjected to a lap shear examination, which contrasted samples that were assembled as a single piece using the Markforged Mark Two 3D printer with samples that were bonded using a two-part Araldite^®^ adhesive. The findings imply that the fatigue life of 3D-printed samples was enhanced using a post-printing platen press and that single-printed samples have a greater strength as compared to adhesively bonded samples. Figure 11 represents the expected compound annual growth rate till 2026, for various industrial sectors concerning the 3D printing of composites. Discussed below are some of the most promising sectors for the FDM of CFRP.

### 3.1. Aerospace Sector

Passenger aircraft are frequently constructed with fiber-reinforced polymer (FRP) composites. CFRPs were used by the Airbus A300 for its airbrakes, rudders, and spoilers. The Airbus A330/340 was constructed using a variety of well-known FRP composites, including carbon FRP, glass FRP, and aramid FRP [9]. Composites are thought to make up about 50% (by weight) of contemporary aircraft. The Boeing 787 was the first airplane to employ composite materials as the main structural element of the airframe construction. In addition, the aircraft carries 23 tons of reinforced materials. Key aircraft body components such as the fuselage, radon, elevators, wing flaps, vertical fins, and horizontal stabilizers are made of FRP composites. Carbon-fiber epoxy is laid out by robotic heads, and the fibers are reinforced in the appropriate directions to support the maximum stresses [146]. The aircraft sector has benefited greatly from 3D printing as it can replace heavy, inflexible, and poorly designed geometric structures with lighter, more flexible alternatives that use less fuel and generate less material waste [147,148]. Drones are currently being tested for short-distance cargo delivery, and as drone technology gains traction, so does its use for automating other sectors of society. The process of 3D-composite printing is being utilized to construct complex parts for the enhanced aerodynamic design that can be manufactured efficiently and that has a high specific strength and stiffness, all to advance the technology. The mechanical and fracture behavior of continuous glass-fiber-reinforced onyx, carbon-fiber-reinforced onyx, and high specific strength, high-temperature polyamide 6 (onyx) composites were reported, along with their 3D printing processes, by Vedrtnam et al. [149]. In comparison to plain onyx samples, the onyx + CF composites showed improvements in Young’s modulus and tensile strength of up to 124% and 134%, respectively.

### 3.2. Defense Sector

Since FRP composites can prototype complicated forms and have high strength and low weight together with corrosion resistance, they are ideal for use in military and defense applications. Following World War II, this class of materials saw extensive use in military and defense applications. The military industry selected them over traditional metals and steels due to their low weight, fatigue resistance, and anti-corrosive properties. Buildings corrode more easily when exposed to salty waterways, which is why this happened. Applications for FRP composites include fighter aircraft, undersea constructions, military vehicles, bunkers, and equipment for fighting wars. These materials were chosen because of their exceptional strength and light weight, as well as their dependable operation and ease of maintenance over time. Security enforcement agencies use protective clothing in which FRP composites are employed [9].

### 3.3. Automobiles Sector

The automotive industry first created more advanced engineered materials to replace traditional metals and alloys to save costs and weight. According to the reports, the weight of cars directly affects 75% of fuel usage [150]. Furthermore, the automobile industry is extremely competitive, and many automotive components are expected to perform better and last longer. Composite materials make up the bulk of an automobile’s load-bearing and structural components, including the body, chassis, hoods, brakes, and electronics [151]. Cast iron was once used to make engine parts for automobiles; however, this had the disadvantage of reducing fuel efficiency and slowing down engine speed. Aluminum alloys are currently being used to replace cast iron components. Since a single material cannot provide all the qualities needed for a successful product, combinations of two or more materials have been used to provide the necessary qualities; these materials are referred to as composite materials, and FRP composites in particular are being used for automobile bodies [150].

### 3.4. Civil and Construction Sector

FRP composites are extensively used in the construction sector. The traditional steels that were once utilized to make reinforcing bars for concrete structures have largely been superseded by them. Fiber-reinforced polymer (FRP) composites are frequently used in the construction industry due to their low weight, high strength, low thermal conductivity, high impact strength, resistance to corrosion, and electromagnetic transparency. They do, however, have several drawbacks specific to the construction sector, including excessive brittleness, poor shear and bending strength, vulnerability to fire, and high initial cost [152]. FRP composites are also widely used in bridge building. Exothermic resin is the primary cause of the curing limitation of FRP composites. Thicker samples limit the heat produced during the curing process, which occasionally results in immediate combustion. Around the world, pultruded profiles made of FRP composites are employed in many bridge structures.

### 3.5. Evolution of Machine Learning-Based AM

The integration of machine learning (ML) into additive manufacturing (AM) marks a significant evolution in the field of manufacturing technology. Recent advancements have seen the application of ML in various aspects of AM, including design optimization, material selection, and process control. For instance, generative design algorithms can create complex structures that are both lightweight and strong, while ML models can predict the optimal combination of process parameters to achieve desired material properties. Moreover, real-time monitoring systems powered by ML can detect and correct defects during the printing process, significantly reducing the rate of failures and improving overall productivity.

The improved uptake of metal powder-bed fusion in industry might be facilitated by overcoming the process and material uncertainty by conducting in situ real-time process–structure–property tests. Every printed layer is being monitored in real-time using thermal and image-based data collection techniques. Although it is computationally expensive, current crystal plasticity finite element (CPFE) modeling can forecast the corresponding strength based on a microstructural picture and material data. This study developed a trained deep neural network (DNN) model that quickly assesses the output, i.e., strength prediction associated with a given input, i.e., the microstructure of multi-phase additive-produced stainless steels using a huge database of input–output samples from CPFE modeling. Phase regions and the corresponding distinct changes in crystallographic orientation are effectively identified by the DNN model. Additionally, because of the different microstructure, it records variations in the macroscopic stress response [153].

Homola et al. [154] discussed the application of machine learning to fatigue life prediction. The initial dataset was predicated on the stress level, fatigue life, and characteristics of defects determined by micro-computed tomography before fatigue testing on additively manufactured Ti6Al-4V samples. Given that the initial dataset was deemed insufficient for training a complete machine learning model, the research presented a unique strategy for augmenting the dataset. Inverse transform sampling and multivariate radial basis function interpolation with different values of the smoothing parameter were used for dataset augmentation. Ultimately, the accuracy of the machine learning model is enhanced to 0.953 coefficient of determination. Homola et al. [155] also presented a framework that utilizes machine learning and Spearman’s rank correlation analysis as an efficient tool to address the impact of stress amplitude and defects identified through micro-computation on the fatigue life performance of AM Ti-6Al-4V. The models of support vector regressor, random forest regressor, and artificial neural network are put into practice and optimized. Using the leave-one-out cross validation method, the hyperparameters and parameters were tuned for the optimization on the training set. The findings validated the suggested framework by comparing the expected and experimental results. The convergence of ML and AM represents a paradigm shift towards more intelligent, autonomous, and efficient manufacturing systems.

## 4. Conclusions and Recommendations

Following a review of the literature, a range of three-dimensional printing techniques employing continuous fiber-reinforced polymers can provide new and enhanced mechanical characteristics of more flexible and lighter structures. The FDM method is the best recommended for the AM of CFRP. Long/continuous fiber can effectively transfer loads; therefore, it provides the greatest performance boost. Although the short fiber reinforcement is more flexible and formable than the continuous, it exhibits a lesser gain in mechanical performance. Even though adding fibers increases performance overall, the inadequate fiber–matrix interaction weakens the filament’s interior structure. The fiber also hinders printed raster fusion, which leaves the printed part porous and with insufficient inter-raster bonding. The printed materials are made of short fiber-reinforced composite materials that have experienced higher longitudinal strength; however, the continuous fibers yield better strength, along with delamination issues that need to be managed.

Summarized below are the conclusions of the processing parameters’ effects, of printing CFRP via FDM, on the performance, as follows:

i. Increasing temperature results in the improvement of the samples’ mechanical characteristics. However, CFRCs lose their dimensional accuracy and appearance at even higher print temperatures.
ii. The mechanical properties of CFRCs are typically enhanced by increasing the fiber volume fraction and infill density while they are reduced by increasing the layer thickness and printing speed.
iii. When the filament feed rate is increased, the mechanical properties first become better and then stay the same.
iv. The optimum mechanical qualities were obtained using the triangular, hexagonal, and rectangular infill designs.
v. The strain to failure of all the composites printed at 0.90 and 0/90/±45 was about the same; however, the ±45° sample’s strain to failure was around four times higher than that of the other samples [113]. As the fiber angle increases, the mechanical characteristics of specimens printed with an isotropic pattern become progressively worse. The optimal mechanical qualities are provided by a 0-degree fiber angle.
vi. Mechanical characteristics were much improved using heated compaction rollers; nevertheless, the samples should not be overly compressed.

Engineering, food, aerospace, construction, automotive, defense, and medical are the industries that have benefited most from the progression of AM. FDM has been increasingly popular compared to additional AM techniques due to cheap initial cost, better reliability, lower maintenance costs, and accessibility to inexpensive materials.

Based upon the conducted literature review, listed below are some of the most promising domains, relevant to the AM of CFRP via FDM, on which future studies could be conducted, as follows:

Most of the research has been performed on particle fibers and short fibers for FDM printing; however, continuous fibers are still in the initial phase and there is considerable capacity to research the 3D printing of CFRP. Owing to the effectiveness and cheap production costs, the 3D printing of CFRP is a novel concept. There is a research gap on the long-term performance of CFRP structures with 3D printing technology. This might be an intriguing subject for future research, as most studies have concentrated on production conditions, and only a small number have focused on post-processing to improve the mechanical characteristics of CFRPs. The critical fiber length, which has a significant impact on the strength of composites, has not received enough attention in the literature that is currently accessible, when it comes to investigations of fiber reinforcing in FDM. The impact of fiber volume fraction on the bending strength of the composite material: The extrusion and mixing process results in the breakage of the fibers. Filament production techniques and mechanisms should be devised for CFRP to be utilized for printing via FDM to yield a suitable fiber length. The build orientation parameter has not been thoroughly studied by researchers, so further research is also necessary on this parameter. Examining methods for aligning fibers before processing filament production: Glass and carbon reinforcements are utilized in most of the research studies; however, there is space to study the performance of other fibers such as Kevlar, onyx, basalt, etc. Subsequent research on composite printing ought to concentrate on decreasing the melt viscosity, refining the nozzle system to obtain the necessary pressure drop and addition of plasticizers to improve the flow. It is recommended to conduct further research studies on fatigue, indentation, wear, creep, dynamic, impact, and friction properties. Most research has focused on composites composed of amorphous low-strength, easy-to-use polymers like PLA and ABS. Optimizing the printing process is suggested for high-performance polymer composites like PEI and PEEK. Hybrid fibers can also be used, and the mechanical properties can be explored. The future research should focus on the usage of recycled thermoplastics and fibers to create products that are less expensive and need less energy to manufacture. Various mechanical properties of printed CFRPs could be tested, and in particular the most common ones (tensile and flexural properties), e.g., impact, compressive, shear strength, and modulus wear and tear in the printed structure. The automated quality inspection of the additively manufactured component is a developing field. It has been observed that machine learning techniques based on artificial intelligence have good scope with respect to AM. However, most research and development are focused on traditional FDM printing for polymers alone. In order to maximize design choices, the 3D-printed item may also be successfully optimized using machine learning-based techniques.

## Figures and Tables

**Table 1:** Studies conducted on different types of fibers and polymers (2019–2024).

Sr.#.	Author Name	Year	Fiber	Polymer	Remarks
1	Antonios et al. [36]	2024	Carbon Fiber	Onyx	The neat onyx material served as a reference while carbon fiber samples with 2, 4, and 6 reinforcing layers out of a constant total layer number of 16 were examined. Micro-X-ray Computed Tomography readings were used to position representative samples. Tensile strength does not increase linearly from 0 to 4 continuous fiber layers; samples reinforced with 6 layers exhibited lower tensile strength than those with 4 layers.
2	Hu et al. [37]		Carbon Fiber	PLA	Many technologies that are now feasible, and their key components depend on the kind of carbon fiber substrate and its structure, have been discussed. The study focuses on the creation of CFRCs made additively with FDM and selective laser sintering (SLS). Furthermore, a thorough explanation of CFRCs made via additive manufacturing was provided.
3	Naik, Thakur and Salunkhe [32]	2023	Glass Fiber	Onyx	The specimen with the triangle infill pattern and 0/90 fiber orientation has a maximum tensile strength of 148.01 MPa, according to the results of the tensile test. In contrast, the drop impact test findings revealed that the triangular infill design with 0/90 fiber orientation absorbs the most impact energy, 8.98% more than the rectangular and honeycomb patterns, respectively.
4	Vatandaş et al. [38]		Carbon Fiber	PLA	The findings show that whereas the fiber bundle size primarily affects flexural and ILSS performance, it has little effect on tensile strength. In ILSS testing, the bundle size impact was much more prominent, with 6K bundle size exhibiting the highest strength.
5	Zhang et al. [21]		Glass Fiber	PA6	The introduction of ultrasonic strengthens the bonding strength between interlayers and inter-filaments, improving the bonding between glass fiber reinforcement and PA6 matrix. Moreover, the mechanical characteristics all improve, and the porosity of printed samples dramatically drops with an increase in ultrasonic frequency, from 4.52% to 1.33%.
6	Ding et al. [39]		Carbon Fiber, Glass Fiber	Nylon	Comparing the impact strength to single carbon fiber-reinforced nylon composites, it climbed to 250% of the original value, while the tensile strength only lost 7% of its original value. With the same fiber content but different layouts, the printed hybrid composites’ tensile and impact strengths varied by 20% and 35%, respectively.
7	Ahmad et al. [40]	2022	Oil Palm Fiber	ABS	Through the Taguchi experiment, the tension and bending strengths of the reinforced material were maximized. Various parameters were tested and the most important printing parameter influencing tensile and flexural behavior was printing orientation.
8	Xiping Li et al. [41]		Carbon Fiber	Nylon PA6	High-strength CF-Nylon composite was produced using a screw-extrusion 3D printer that was uniquely designed. The porosity and fluidity of the composites were reduced by the inclusion of carbon fiber.
9	Ziyan Man et al. [42]		Carbon Fiber	Nylon	Three factors: fiber/matrix bonding, fiber orientation, and fiber distribution affect the scratch behavior of 3D-printed CF-PA6. The three main processes of wear are loss of fiber, breaking of fiber, and abrasion.
10	Müller et al. [43]		Bamboo Pinewood Cork	PLA	PLA composites and 3D-printed PLA were evaluated for low cycle fatigue. When compared to pure PLA, all composites exhibit reduced tensile and fatigue characteristics.
11	Uşun and Gümrük [31]	2021	Carbon Fiber	PLA	The melt impregnation line was used to produce the CFRTP filaments internally. Comparing CFRTP composites with 22% and 33% CF, the 40% CF composites exhibited superior tensile and flexural strength.
12	Joel Galos et al. [44]		Carbon Fiber	Nylon	In contrast to a hot-molded composite composed of nylon reinforced with carbon fiber, FDM 3D-printed material had reduced longitudinal electrical conductivity. In comparison to molded composites, 3D-printed composites offer superior electrical conductivities through and transverse-thickness.
13	Garofalo and Walczyk [45]		Carbon Fiber	LDPE Nylon Polycarbonate	A new production method was developed to produce CFRP. Although the created filament had a higher volume fraction and pre-preg quality, it is unknown what effects the new filament has on the mechanical properties.
14	Prajapati, Dave and Raval [46]		Glass Fiber	Onyx (Nylon + Chopped Carbon fiber),	The addition of additional glass fiber reinforcing layers to the composite boosted its impact strength.
15	Bhagia et al. [47]	2020	Poplar wood	PLA	Examination of two poplar-PLA composites (15% fibrillated poplar and 20% milled poplar) with respect to tensile testing. In terms of tensile strength, neat PLA outperforms both poplar wood-PLA composites.
16	Wang et al. [48]		Carbon Fiber, Glass Fiber	PEEK	The interfacial bonding between GF/PEEK and CF/PEEK is superior. Both composites have greater mechanical strengths (tensile, flexural, and impact) in comparison to neat. The mechanically strongest composite materials are those that include 5% weight fiber. Strengths were decreased when the fiber content was raised from 5% to 15%.
17	García et al. [35]		Graphene	PLA	The results showed that dimensional accuracy was mostly affected by the construction orientation, with an increase in layer area on the X-Y plane. The Z-axis dimensional accuracy was essentially typical, with no variation from the accumulation of layers or any printing parameter. Building orientation had a significant influence, with flat orientation yielding the best results.
18	Ming et al. [14]		Glass Fiber	Epoxy	3D-printed CGF/EP samples with a 43 ± 3 weight percent fiber content demonstrated yield strength and modulus of elasticity of 272.51 ± 5.12 MPa and 8.01 ± 0.45 GPa, respectively, flexural strength and modulus of flexural modulus of 299.36 ± 6.16 MPa and 8.35 ± 0.18 GPa, and interlaminar shear strength of 34.06 ± 0.83 MPa.
19	Zhang et al. [49]	2019	Carbon Fiber	PLA Nylon	The tensile and bending strengths of CCF-PLA are higher than those of clean PLA and short carbon PLA composite. Likewise, continuous carbon fiber-nylon gave improved results in terms of flexural and yield strength than neat nylon polymer.
20	Mohammadizadeh et al. [50]		Carbon Fiber, Glass Fiber, Kevlar	Nylon	All composites’ tensile, fatigue, and creep properties were examined. Kevlar- and GF-reinforced composites fared worse than carbon fiber-reinforced composites. Three factors were shown to be responsible for the failure of fiber-reinforced nylon: pullout, breaking, and delamination.
21	Mei, Ali, Yan [51]		Carbon Fiber	Nylon	Compared to samples printed at fiber angles [30°/45°/60°] and [15°/45°/75°], the sample created with mixed isotropic fiber angle [0°/45°/90°] was stronger. Non-hot-pressed composites, in comparison to hot-pressed composites, have a greater modulus and tensile strength.
22	Chabaud et al. [52]		Carbon Fiber, Glass Fiber	PA	In every printing pattern or printing intensity, onyx samples outperformed pristine nylon in terms of Young’s modulus and yield strength. The quantity of fiber in the carbon fiber–nylon matrix enhanced its tensile qualities.

**Table 2:** The 3D printing techniques with material type segregation.

3D Printing Techniques			
Technique Name	Powder	Liquid	Solid
Selective Laser Sintering	Yes	No	No
Stereolithography	No	Yes	No
Fused Deposition Modelling	No	No	Yes
Selective Laser Melting	Yes	No	No
Direct Metal Laser Sintering	Yes	No	No
Solid Ground Curing	No	Yes	No
Robocasting	No	No	Yes
Direct Metal Deposition	Yes	No	No
Laser Transfer Printing	No	Yes	No
Laminated Object Manufacturing	No	No	Yes
Smog Formation Potential	No	No	Yes
Thermojet	No	Yes	No
Digital Light Manufacturing	Yes	No	No
Multi Jet Modelling	No	Yes	No
